# S236. IS MAINTENANCE TREATMENT NEEDED WHEN THE FIRST EPISODE OF PSYCHOSIS IS NOT DUE TO SCHIZOPHRENIA?

**DOI:** 10.1093/schbul/sby018.1023

**Published:** 2018-04-01

**Authors:** Gbolahan Odejayi, Robert Zipursky

**Affiliations:** McMaster University

## Abstract

**Background:**

Debate continues about how long maintenance treatment should be continued following a first episode of psychosis (FEP). Resolving this question requires an understanding of the risk of recurrence which would be expected to vary as a function of the underlying cause of the psychosis. The range of diagnoses that may present as a FEP include schizophrenia and related schizophrenia spectrum disorders, bipolar mania, bipolar and unipolar depression, substance-induced psychosis, and unspecified psychotic disorders. The majority of FEP patients will receive the diagnosis of schizophrenia or bipolar disorder for which the 1- year risk of illness recurrence is estimated at 77% and 41%, respectively. We reviewed the literature in order to estimate the risk of relapse and the risk of developing a primary psychotic disorder following a FEP due to other diagnoses.

**Methods:**

We conducted a primary literature review using Medline and PubMed. We included the following search terms: first episode, relapse, recurrence, depression with psychosis, psychotic depression, mania with psychosis, substance induced psychosis and psychosis. We included prospective and retrospective studies including those that involved medication discontinuation or naturalistic follow-up to determine the risk of recurrence following a FEP. We also reviewed the literature to determine the likelihood that FEP with these diagnoses would transition to a primary psychotic disorder (schizophrenia spectrum disorder or major mood disorder) for which published rates of recurrence would apply.

**Results:**

Two studies were identified which reported on the recurrence rate following a first episode of psychotic depression. Recurrence rates ranged from 27% at eight months to 80.6% at a mean of 32 months. An additional study found that following a first episode of psychotic depression, 29.9% and 14.3% of patients were diagnosed with schizophrenia and bipolar disorder, respectively, at 10-year follow-up. The risk of developing a primary psychotic disorder following a first episode of substance-induced psychosis has been investigated in three studies which reported rates of conversion to a primary psychotic disorder of 25% at one year, 25% at 10 years and 32% at 20 years. The risk of developing a primary psychotic disorder following a cannabis-induced psychosis has been investigated in three studies which reported rates of conversion of 44.5% at three years, 46% at eight years, and 47.4% at 20 years. Patients with a first episode of unspecified psychosis have been reported in a single study to have a 73.7% risk of developing a primary psychotic disorder at 10 year follow-up.

**Discussion:**

The risk of illness recurrence following a FEP not initially diagnosed as a schizophrenia spectrum or bipolar disorder was found to vary by both initial diagnosis and by follow-up duration. Psychotic depression, substance-induced psychosis and other unspecified psychoses were all associated with either substantial risks of illness recurrence or development of a primary psychotic disorder. The risk of illness recurrence following medication discontinuation has not been established for these disorders as many of these studies included patients whether they were on or off of their prescribed medications. Clinical recommendation should be informed by future research on recurrence rates with and without maintenance medication for the different causes of FEP. In the meantime, patients with a FEP and their family members should be fully informed about the risk of illness recurrence and development of a primary psychotic disorder when considering any trial of medication discontinuation.

